# Adsorption of Toluene and Water over Cationic-Exchanged Y Zeolites: A DFT Exploration

**DOI:** 10.3390/molecules26185486

**Published:** 2021-09-09

**Authors:** Etienne P. Hessou, Lucie A. Bédé, Hicham Jabraoui, Abderrahmane Semmeq, Michael Badawi, Valentin Valtchev

**Affiliations:** 1 Laboratoire de Physique et Chimie Théoriques, Faculté des Sciences et Technologies, CNRS, Université de Lorraine, Boulevard des Aiguillettes, 54500 Vandoeuvre-lès-Nancy, France; abderrahmane.semmeq@univ-lorraine.fr (A.S.); michael.badawi@univ-lorraine.fr (M.B.); 2 Laboratoire de Constitution et Réaction de la Matière, Université Felix Houphouët-Boigny, 22 BP 582 Abidjan 22, Côte d’Ivoire; lucie.bede@univ-fhb.edu.ci; 3 Université Paris-Saclay, CEA, CNRS, NIMBE, 91191 Gif-sur-Yvette, France; hicham.jabraoui@cea.fr; 4 Laboratoire Catalyse et Spectrochimie, Normandie Université, ENSICAEN, CNRS, 6 Boulevard Maréchal Juin, 14050 Caen, France; valentin.valtchev@ensicaen.fr

**Keywords:** adsorption, toluene, DFT, zeolite Y

## Abstract

In this study, density functional theory (DFT) calculations have been performed to investigate the adsorption mechanisms of toluene and water onto various cationic forms of Y zeolite (LiY, NaY, KY, CsY, CuY and AgY). Our computational investigation revealed that toluene is mainly adsorbed via π–interactions on alkalis exchanged Y zeolites, where the adsorbed toluene moiety interacts with a single cation for all cases with the exception of CsY, where two cations can simultaneously contribute to the adsorption of the toluene, hence leading to the highest interaction observed among the series. Furthermore, we find that the interaction energies of toluene increase while moving down in the alkaline series where interaction energies are 87.8, 105.5, 97.8, and 114.4 kJ/mol for LiY, NaY, KY and CsY, respectively. For zeolites based on transition metals (CuY and AgY), our calculations reveal a different adsorption mode where only one cation interacts with toluene through two carbon atoms of the aromatic ring with interaction energies of 147.0 and 131.5 kJ/mol for CuY and AgY, respectively. More importantly, we show that water presents no inhibitory effect on the adsorption of toluene, where interaction energies of this latter were 10 kJ/mol (LiY) to 47 kJ/mol (CsY) higher than those of water. Our results point out that LiY would be less efficient for the toluene/water separation while CuY, AgY and CsY would be the ideal candidates for this application.

## 1. Introduction

Volatile organic compounds (VOCs) are major air pollutants that are harmful to human health and to the environment [1,2,3,4]. These pollutants affect air quality and have an impact on global warming [5,6,7,8]. Among them, toluene is one of the worst pollutants classified by the Environmental Protection Agency of the United States due to its toxicity and significant emissions [9]. Toluene is used in many industrial products such as paints, thinners, glues, adhesives, cleaning products, and so forth [10]. Exposure or inhalation of toluene vapors can cause health issues such as neurotoxicity, cardiopulmonary problems, atrophy, encephalopathy, and eye irritation [11,12,13,14,15,16,17,18]. The severity of the symptoms is often correlated with the concentration of toluene vapor. It has been shown that exposure to high concentrations of toluene (>45,000 mg/m^3^) for about 12 h can be fatal [19,20]. Besides its toxic activity, toluene is also considered a biomarker [21,22,23]; abnormally high concentrations of toluene can be found in the breath of patients with lung cancer. Therefore, toluene sensors can be very helpful for the early-stage diagnosis of lung cancer.

Given the impact of toluene on the environment and on human health, several experimental and theoretical studies have focused on reducing the level of this pollutant using various adsorption methods [24,25,26,27,28,29,30]. One of the most promising techniques for removing VOCs from indoor or outdoor environments is to trap them in inorganic adsorbents such as zeolites [26,31,32,33,34]. These materials have been the subject of many applications in adsorption and catalysis processes due to their unique properties, such as the ability to exchange cations, a high specific surface area and a relatively inexpensive production process [35]. In addition, they have good performance in terms of VOC adsorption capacity, good thermal stability, and good selectivity. These various properties make zeolites extremely popular materials for the adsorption of VOCs [31,36]. Among the 200 types of existing zeolites [36,37], mordenite (MOR), ZSM-5 (MFI) and faujasite (FAU) are the most used zeolites in the treatment of VOCs [26,33,34]. In addition, they also find applications in biomedical fields. Toluene sensors for lung cancer detection require a concentration step prior to detection; adsorbing the molecule in highly porous material, such as zeolites, greatly improves these sensors [21].

In particular, faujasite zeolites are very often used in the separation of aromatics and hydrocarbons in petroleum processing due to the size of their pores going up to 7.4 Å [38]. They have been synthesized and exchanged with many different extra framework cations such as Na^+^, K^+^, Rb^+^, Cs^+^, Ag^+^, Ca^2+^ and Ba^2+^ [39,40]. In addition, faujasites are often used as a molecular sieve, an application that exploits their adsorption properties, and in the selective catalytic reduction process [26,33,41]. Accordingly, for many years now, faujasites have been used in the refining industry as the catalytically active commponent in cracking catalysts [42,43,44]. Previous works on the adsorption of xylene on faujasite have shown that the selectivity towards different mesomers of this molecule can be linked to the nature of cations [45,46,47]. Deng et al. [48] performed experimental and *ab initio* adsorption studies to compare the toluene adsorption capacity on FAU, BEA, and MFI zeolites where they reported superior selectivity of FAU USY towards toluene compared to water and dichloromethane. While Serra et al. [49,50] investigated the adsorption of toluene on different zeolite samples with various Si/Al ratios that were modified with varying loadings of exchangeable cations (Na^+^, H^+^ or Cs^+^). They showed that the thermal stability of the adsorbed toluene was mostly related to the type of the exchanged cation, which controls the overall basicity, rather than the structure of the zeolite. It arises that the most important factor is indeed the chemical nature of the chosen cations.

Wang and co-authors [51] conducted an experimental study to investigate the capture of VOCs by MFI-type zeolites in humid conditions, and they reported the competitive adsorption of toluene and water vapor. Liu et al. [52] showed that the presence of water could substantially influence the adsorption of toluene, stressing the importance of selective adsorbents of VOCs. Indeed, ambient air is a carrier gas, where a typical relative humidity (hr) of 60% corresponds to a water content of approximately 12.3 gm^−3^ or 1.66 vol-% at room temperature (T = 23 °C) [53]. However, to date, studies on VOCs–water mixtures’ adsorption remain scarce [48].

In this study, we investigate the adsorption mechanisms of toluene and water in some monovalent cation exchanged Y zeolites; simultaneously, we systematically identify the cations that give the best adsorption capacity for the target molecule (i.e., toluene). We use state-of-the-art Density Functional Theory (DFT) calculations, including dispersion corrections. This approach has been proven to provide reliable results to elucidate the mechanisms of adsorption in zeolite materials at the atomistic level [54,55,56,57]. In fact, nowadays, the chemical accuracy of DFT simulations is high enough to predict the enthalpies of the adsorption of various molecules in zeolites, which agrees remarkably with the data obtained in calorimetric experiments [55,56,57,58,59]. Periodic DFT approaches have also been used to study the capture of various pollutants such as carbon dioxide, methane [60], or iodine species from nuclear streams [61,62,63].

The paper is organized as follows: we present and discuss the results obtained for the type and strength of interactions between toluene/water and FAU Y exchanged with Li^+^, Na^+^, K^+^, Cs^+^, Cu^+^ and Ag^+^ cations. Then, a section dedicated to the calculation procedure is presented. The main conclusions drawn from this work are given in the last section.

## 2. Results

Using the DFT methodology as detailed in Section 3, we investigated the adsorption of toluene and water on different cation-exchanged Y faujasites. The preferential adsorption modes, as well as the interaction energies, of both molecules (toluene and water) over LiY, NaY, KY, CsY, CuY and AgY are presented and discussed below. The CONTAR files of most stable configuration calculated for toluene and water on different cation-exchanged Y faujasites are available in supplementary materials.

### 2.1. Toluene Adsorption

**Figure 1:**
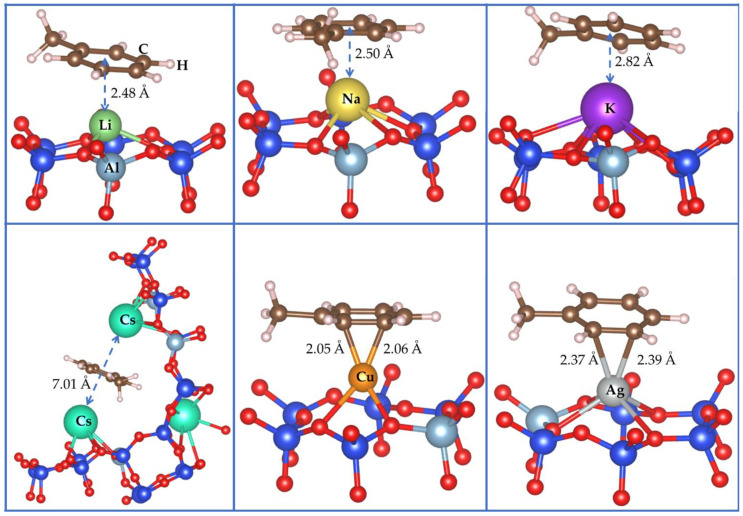
The most stable adsorption modes calculated for toluene over LiY, NaY, KY, CsY, CuY and AgY. For the sake of clarity, only the 6-MR involved in the adsorption process was displayed (One 6-MR of LiY, NaY, KY, CuY and AgY; two 6-MR for CsY).

As can be seen from Figure 1, where we presented the most stable configurations of adsorbed toluene, the flat adsorption is the most stable mode found for toluene molecules adsorbed onto alkaline exchanged zeolites. The preference of this layout is explained by the fact that it allows π–interactions, which are known to be the most favorable adsorption mode between aromatic rings and cations in zeolites [64,65,66]. In addition, with alkalis, the cation acts as Lewis acid and, hence, it is expected to polarize the electron cloud of the aromatic ring [27]. Toluene is then adsorbed onto LiY, NaY and KY over one cation of Li, Na and K, respectively.

In the case of LiY, the carbon atoms of the aromatic ring are located in the range of 2.57–3.12 Å (in average 2.84 Å) with respect to Li^+^, while the aromatic center is about 2.48 Å of the Li^+^ (see Table 1). In the case of NaY, the calculated distance between carbon atoms of the aromatic ring of the toluene and the interacting Na^+^ cation are in the range of 2.82–2.95 Å (in average 2.87 Å), and the aromatic center is about 2.5 Å of the Na^+^ cation. For KY, the distances (K to the carbon of the aromatic ring or K to the aromatic center) are respectively 3.10–3.20 Å and 2.82 Å. One can observe that the increase of these average distances is proportional to increasing ionic radii of Li^+^, Na^+^ and K^+^ and, as can be expected, this same observation naturally extends to Cs^+^. In fact, in CsY, the increasing of the ionic radius allows the toluene molecule to interact with two Cs cations simultaneously while it only interacts with a single cation in the case of LiY, NaY, and KY. Toluene establishes a π–interaction with both Cs cations, where the average distance between the first Cs cation and the carbon atoms of the aromatic ring is 3.88 Å. The second cesium, diametrically opposed to the first, interacts with toluene at a distance of 3.4 Å from the aromatic center of toluene and an average of 3.68 Å from the carbon atoms of the aromatic ring. 

**Figure 2:**
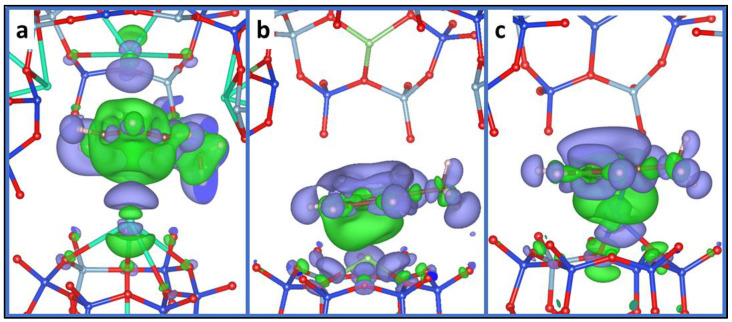
The difference in electron density (Δ*ρ*) induced by the adsorption of toluene in CsY (**a**), LiY (**b**), and AgY (**c**) faujasites, respectively. The blue (green) zones indicate density increase (decrease). Where cyan balls represent Cs, light green balls represent Li, grey balls represent Ag, blue balls represent Si, and red balls represent O.

The calculation of the electron density profiles confirms the geometrical analysis; where the double contribution of the Cs^+^ cations is clearly shown through the electron density map (see Figure 2a). This observation is totally different from what we found for Li^+^, Na^+^ and K^+^ cations, where the computed electron density shows that there is only one cation that can contribute to the adsorption of toluene in FAU, due to a shorter radius of these cations.

The interaction energies of toluene with LiY, NaY, KY and CsY are 87.8/105.5/97.8/114.4 kJ/mol, respectively (see Table 2). An increase of the interaction energy is observed as we go down in the alkaline series (Li to Cs) since the electronegativity decreases considerably from Na to Cs. Although one would expect that the interaction energy of toluene with CsY would be weaker than that with NaY, the possibility of bidentate interaction explains the fact that toluene is 10 kJ/mol more adsorbed on CsY than NaY. A previous study of benzene adsorption on faujasite Y exhibits the same energy trends [67].

Furthermore, we investigate the adsorption of toluene with CuY and AgY where transition metallic cations are embedded. The distances between the cation and the aromatic ring vary from 2.05 to 3.47 Å for CuY and from 2.37 to 3.57 Å in the case of AgY. Unlike alkaline cations, the interaction of toluene is established rather by two of the carbon atoms of the aromatic ring. The two distances M–C (M = Cu and Ag) are 2.05/2.06 Å for CuY and 2.37/2.38 Å for AgY. The interaction energies of toluene are 147.0/131.5 kJ/mol, respectively, for CuY and AgY. These two systems give the highest interaction energy of toluene. Here, we can see that only transition metal cation that can contribute to the adsorption of toluene in FAU. This is supported by the electron density map in which the charge of the second metal was not affected by the adsorption of toluene, evidencing the absence of any interaction (see Figure 2c).

### 2.2. Water Adsorption

**Figure 3:**
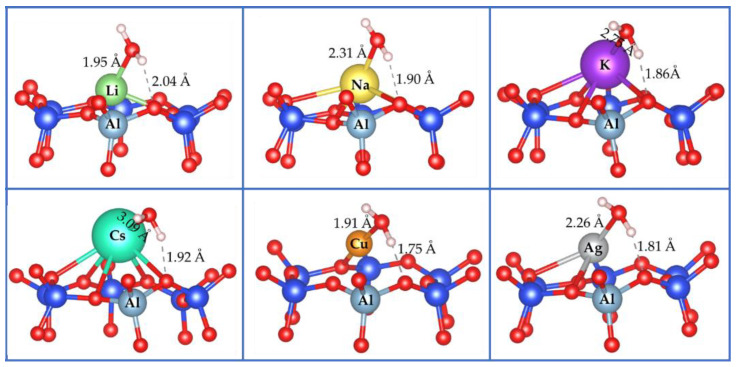
The most stable adsorption modes calculated for water over LiY, NaY, KY, CsY, CuY and AgY. For the sake of clarity, only the 6-MR involved in the adsorption process was displayed. Color codes: silicon in blue and oxygen in red.

Adsorption of water has been investigated on LiY, NaY, KY, CsY, CuY, and AgY. The most stable adsorption modes are displayed in Figure 3. We can see that the preferential adsorption mode of water is through its oxygen atom for all cases. In the case of systems with alkali metals, LY, NaY, KY, and CsY, the M–O distance between the metal and oxygen atom of water is 1.95/2.31/2.75/3.09 Å, respectively. This distance increases in proportion to the increase in the ionic radius from Li to Cs. A hydrogen bond is established during water adsorption between one hydrogen atom of water and the nearest oxygen atom of the 6MR. The H-bond distances are 2.04/1.90/1.86/1.92 Å, respectively, for LiY, NaY, KY, and CsY.

The interaction energy calculated for water is 74.3 kJ/mol in LiY, 70.2 kJ/mol in NaY, 68.8 kJ/mol in KY, and 67.3 kJ/mol in CsY (see Table 2). The highest interaction is obtained with LiY, and the smallest with CsY. These results are in agreement with experimental ones, as Dzhigit et al. [68] reported that the adsorption enthalpy of water at 23 °C is about 67 kJ/mol in LiNaY (44Li, 6Na); 66 kJ/mol in NaY and about 65 kJ/mol in KNaY (55 K, 2Na). The interaction energy decreases slightly from Li to Cs in line with the decrease in electronegativity along the alkali metal series. This observation can be explained by the HSAB theory of Pearson [69,70,71] and was described in our previous work with a higher Si/Al ratio [72,73]. In addition, the experimental work of Jentys et al. [74] has shown that in alkali-metal-exchanged ZSM-5, the adsorption of water molecules at low coverage on alkali-metal cations are more favored for Li-, Na-, and K-ZSM5 than for Cs- and Rb-ZSM5.

In the case of CuY and AgY, the interactions of the metallic atom (M) and the oxygen atom in water are characterized with M–O distances of 1.91 Å and 2.26 Å, respectively. In comparison, one water H atom is involved in a hydrogen bond with an oxygen atom of the 6MR at 1.75 Å and 1.81 Å for CuY and AgY, respectively. The interaction energy trends show that both structures yield similar adsorption behavior where interaction energies are 79.1 kJ/mol and 76.6 kJ/mol for CuY and AgY, respectively. In previous work, we have found a similar trend with an Si/Al ratio of 47 [72], where water adsorption underlines close interaction energies with an only slight preference for CuY and AgY compared to alkali ones (LiY, NaY, KY and CsY). We point out that this contrasts with the results reported by Kumar et al. [67]. Their damped Car–Parrinello molecular dynamics results showed that the adsorption energies of water in CuY and AgY are about 65 and 70 kJ/mol, respectively. According to their results, AgY is expected to adsorb water more efficiently than CuY. However, these differences remain close to the expected chemical accuracy of the employed methods.

## 3. Discussion and Conclusions 

In the previous sections, we have presented individual details on the adsorption of toluene (Section 2.1) and water (Section 2.2) in some cationic-exchanged forms of zeolite Y (LiY, NaY, KY, CsY, CuY and AgY). At the base of these results, it is now necessary to be interested in the probable effect of water on the adsorption of the target molecule (toluene).

**Figure 4:**
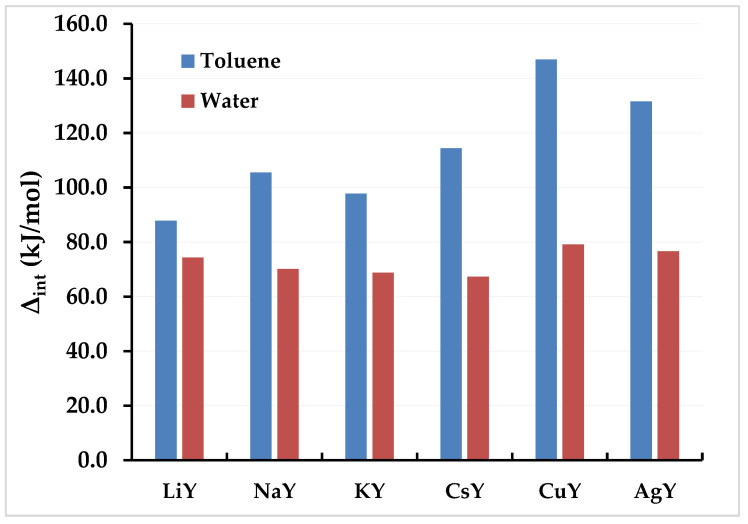
Variation of the interaction energy (kJ/mol) of toluene and water depending on the adsorbent LiY, NaY, KY, CsY, CuY, and AgY.

As shown in Table 2 and Figure 4, toluene is more adsorbed than water on all the systems studied. However, the energy gap between the adsorption of toluene and that of water is a crucial factor that may well inform the choice of the systems.

Threfore, by comparing the interaction energies calculated for toluene and water, the following observations can be made:

- LiY adsorbs more toluene than water with a difference of more than 13 kJ/mol;
- NaY adsorbs more toluene than water with a difference of more than 35 kJ/mol;
- KY adsorbs toluene a little less than NaY, but the toluene/water difference is 29kJ/mol;
- CsY adsorbs toluene more than water with a difference of 47 kJ/mol, thus putting CsY at the top of the list of Y zeolites exchanged for alkali cations;
- CuY and AgY adsorb toluene more than water with 68 and 58 kJ/mol, respectively.

One can therefore classify the investigated systems as follows, CuY > AgY > CsY > NaY > KY > LiY, and conclude that CuY, AgY, CsY and KY are, in decreasing order, the best options for effective selectivity in the adsorption of toluene in the presence of water vapor.

In order to provide a fundamental understanding governing the interaction between toluene/water and several formulations of FAU, we limit ourselves only to the adsorption of individual molecules without considering the impact of one molecule on another. This opens new perspectives for in-depth studies on the adsorption of these two molecules, including experimental investigations or co-adsorption molecular dynamics simulations. 

## 4. Computational Methods

### 4.1. Computation Details

We investigated the toluene and water adsorption onto Y zeolites using the Vienna Ab-initio Simulation Package (VASP) [75]. DFT calculations employed the PBE functional and projector augmented plane wave (PAW) method [76,77] with plane-wave cutoff energy of 450 eV. Kohn–Sham equations were solved iteratively until the energy difference between cycles became lower than 10^−6^ eV, while Gaussian smearing was fixed to 0.1 eV. The Brillouin zone was sampled at the Γ-point only. FAU structures were fully optimized until all the forces fell below 0.01 eV/Å per atom. In order to account for the van der Waals (vdW) interactions, the TS/HI method [78] was used. The TS/HI scheme is an improved version of the TS (Tkatchenko–Scheffler) [79] dispersion correction; it is based on an Iterative Hirshfeld partitioning leading to an accurate description of ionic solids such as cationic zeolites [78,79,80].

To describe the adsorption phenomena, the interaction energy between the molecule (Toluene/Water) and the Y zeolite formulations were evaluated at 0 K using the following equation [67,81]:

(1) $$\Delta E_{int}=\;E_{zeolite}+\;E_{molecule}-E_{zeolite-molecule}$$

where *E_zeolite-molecule_* is the energy of the Y zeolite with adsorbed molecules, *E_zeolite_* is the energy of the empty zeolite, and *E_molecule_* is the energy of the isolated molecule in the gaseous phase. According to this equation, a positive value of Δ*E_int_* corresponds to an exothermic process.

In addition to adsorption investigations in terms of the total energy interaction, we are using the charge density difference (Δ*ρ*) to further enhance our understanding of the adsorption of molecules into the considered faujasite formulations. To visualize the charge density difference (Δ*ρ*) introduced by the adsorption of toluene, we combine the three charge densities: (1) the density of the complex (*ρ_Fau-molecule_*); (2) the density of the isolated molecule (*ρ_molecule_*); and (3) the density of the clean faujasite *ρ_FAU_*, which is formulated as follows:

(2) $$\Delta \rho =\rho_{FAU-molecule}\;-\;\rho_{FAU}\;-\;\rho_{molecule}.$$

### 4.2. Structural Model

The siliceous structure of faujasite crystallizes within the Fd3m symmetry space group [82]. The FAU-type framework consists of cuboctahedral sodalite units connected by hexagonal prisms (D6R), forming large empty cavities called supercages. These supercages are interconnected by their 12-membered ring windows, also called hexagonal windows (12MR), thereby forming the porous network. The standard cell has a cubic structure with the lattice parameters a = b = c = 25.028 Å (576 atoms, Si_192_O_384_) and α = β = γ = 90° [83,84]. However, to reduce the computational effort, we have used a primitive rhombohedral cell (two supercages and eight hexagonal windows connecting the sodalite cages to the supercages) containing 144 atoms. In this structure, 14 atoms of silicon Si(+IV) are replaced by 14 atoms of aluminum Al(+III) to thus obtain a Y faujasite with an Si/Al ratio equal to 2.43. In order to once again reach the neutrality of the system lost due to the introduction of Al(+III), 14 monovalent cations of the same atom are added to obtain LiY, NaY, KY, CsY, CuY and AgY, respectively.

## Figures and Tables

**Table 1:** Distance between the aromatic ring of toluene and sorbents upon adsorption. C corresponds to the aromatic center of toluene; M = Li, Na, K, Cs1, Cs2, Cu, and Ag.

	LiY	NaY	KY	CsY		CuY	AgY
**M**–**C1**	3.125	2.949	3.192	3.706	3.998	2.859	2.385
**M**–**C2**	3.095	2.896	3.132	3.729	3.840	2.052	3.045
**M**–**C3**	2.837	2.841	3.101	3.709	3.738	2.064	2.370
**M**–**C4**	2.569	2.821	3.116	3.657	3.782	2.866	3.566
**M**–**C5**	2.566	2.834	3.156	3.620	3.918	3.474	3.023
**M**–**C6**	2.844	2.891	3.189	3.639	4.020	3.460	3.576
**M**–**C**	2.478	2.504	2.819	3.396	3.619	2.481	2.684

**Table 2:** Calculated interaction energies (kJ/mol) of toluene and water onto LiY, NaY, KY, CsY, CuY and AgY.

	Δ*E_int_* (kJ/mol)	
	Toluene	Water
**LiY**	87.8	74.3
**NaY**	105.5	70.2
**KY**	97.8	68.8
**CsY**	114.4	67.3
**CuY**	147.0	79.1
**AgY**	131.5	76.6

## Data Availability

The data presented in this study are available in supplementary material.

## Supplementary Materials

The CONTCAR files are available online.

## Sample Availability

Zeolite models are available in Supplementary Materials.
